# Genotyping and clinical factors in pediatric diarrhea caused by rotaviruses: one-year surveillance in Surabaya, Indonesia

**DOI:** 10.1186/s13099-015-0048-2

**Published:** 2015-02-08

**Authors:** Subijanto Marto Sudarmo, Katsumi Shigemura, Alpha Fardah Athiyyah, Kayo Osawa, Oktavian Prasetia Wardana, Andy Darma, Reza Ranuh, Dadik Raharjo, Soichi Arakawa, Masato Fujisawa, Toshiro Shirakawa

**Affiliations:** Department of Child Health, Soetomo Hospital, Airlangga University, Surabaya, Indonesia; Indonesia-Japan Collaborative Research Center for Emerging and Re-emerging Infectious Diseases, Institute of Tropical Disease, Airlangga University, Surabaya, Indonesia; Department of Urology, Kobe University Graduate School of Medicine, Kobe, Japan; Division of Infectious Diseases, Department of International Health, Kobe University Graduate School of Health Science, Kobe, Japan; Department of Infection Control and Prevention, Kobe University Hospital, Kobe, Japan; Institute of Tropical Disease, Airlangga University, Surabaya, Indonesia; Center for Infectious Diseases, Kobe University Graduate School of Medicine, 7-5-1, Kusunoki-cho, Chuo-ku, Kobe, 650-0017 Japan

**Keywords:** Genotyping, Clinical factors, Rotavirus diarrhea, Pediatrics

## Abstract

**Background:**

Rotavirus infections are a major cause of diarrhea in children in both developed and developing countries. Rotavirus genetics, patient immunity, and environmental factors are thought to be related to the severity of acute diarrhea due to rotavirus in infants and young children. The objective of this study was to provide a correlation between rotavirus genotypes, clinical factors and degree of severity of acute diarrhea in children under 5 years old in Surabaya, Indonesia.

**Methods:**

A cross-sectional study was conducted in children aged 1–60 months with acute diarrhea hospitalized in Soetomo Hospital, Surabaya, Indonesia from April to December 2013. Rotavirus in stool specimens was identified by ELISA and genotyping (G-type and P-type) using multiplex reverse transcription PCR. Severity was measured using the Ruuska and Vesikari scoring system. The clinical factors were investigated included patient’s age (months), hydration, antibiotic administration, nutritional state, co-bacterial infection and co-viral infection.

**Results:**

A total of 88 children met the criteria; 80.7% were aged 6–24 months, watery diarrhea was the most common type (77.3%) and 73.6% of the subjects were co-infected with bacteria, of which pathogenic *Escherichia coli* was the most common (42.5%). The predominant VP7 genotyping (G-type) was G2 (31.8%) and that of VP4 genotyping (P-type) was P[4] (31.8%). The predominant rotavirus genotype was G2P[4] (19.3%); G1P[4] and G9P[4] were uncommon with a prevalence of 4.5%. There were significant differences between the common genotype and uncommon genotype with respect to the total severity score of diarrhea (p <0.05). G3, G4 and G9 were significantly correlated with severe diarrhea (p = 0.009) in multivariate analyses and with frequency of diarrhea (>10 times a day) (p = 0.045) in univariate analyses, but there was no significant correlation between P typing and severity of diarrhea. For combination genotyping of G and P, G2P[4] was significantly correlated with severe diarrhea in multivariate analyses (p = 0.029).

**Conclusions:**

There is a correlation between rotavirus genotype and severity of acute diarrhea in children. Genotype G2P[4] has the highest prevalence. G3, G4, G9 and G2P[4] combination genotype were found to be associated with severe diarrhea.

## Introduction

Pediatric diarrhea is often fatal since this disease results in severe dehydration [1]. There are several causes of pediatric diarrhea including bacterial or viral infectious diseases. As to the latter, several pathogens such as rotavirus, adenovirus and norovirus are reported [2-4]. Rotavirus is the most commonly isolated viral cause of severe diarrhea in infants, infecting more than 111 million pediatric patients every year and causing an estimated 440,000 deaths [4-6]. Rotavirus genetic factors, patient’s immune factors, and environmental factors are associated with the incidence and severity of acute diarrhea due to rotavirus in infants and toddlers [7].

Viral typing is necessary for characterizing rotavirus strains, especially focusing on different rotavirus seasons in different locations. G-typing is categorized as G1-16, the double combination of G1-16G1-16, and the triple combination G1-16G1-16G1-16 [8]. P-typing is classified to into P1-28, the double combination of P1-28P1-28, and triple combination of P1-28P1-28P1-28 [8]. Moreover, whole genotyping is sometimes shown as the combination of G type and P type [8]. VP7 and VP4 proteins are the basis of the classification of group A rotaviruses by G (VP7) and P (VP4) types; G represents glycoprotein and P represents protease sensitive protein [8,9]. G1P[8], G2P[4], G3P[8], and G4P[8] were the four dominant and most commonly found genotypes worldwide from 1994 to 2003 [10,11]. Generally, in human rotavirus, G1 to G4 and G9 in VP7 and P[4] and P[8] in VP4 are the most common types. Combined G and P typing represents a limited number of genotypes such as G2P[4], G3P[8], G4P[8], G9P[8], and G1P[8] [11]. Those types vary from region to region (e.g. G5 types in Brazil, G10 types in India) [12,13]. Till date 27 G genotypes, 37 P genotypes and 73 different combinations of G and P genotypes have been reported [14,15]. Putnam *et al.* found G9P [8] to be the most common genotype with a prevalence of 13.57% in 2003–2004 in Indonesia, but Soenarto *et al.* detected G1P[6] as the dominant genotype (34%) in Indonesia in 2006 [16,17]. New rotavirus genotypes have been found, causing more severe diarrhea than previously identified rotavirus genotypes [18-23].

Flores *et al.* studying the efficacy of rotavirus vaccine (RIT 4237) on diarrheal disease, formulated a scoring system for the severity of diarrhea in infants and toddlers with or without vaccination [24]. The scoring system was used by Cascio *et al.* to assess differences in the severity of diarrhea experienced by children in Italy who were infected by rotavirus of different strains [19]. Ruuska and Vesikari [22] developed the scoring system by combining more variables includes episodes of vomiting in 24 hours [25]. Its validity and reliability was verified with a significant correlation between scores and the impact of diarrheal disease on the family [26]. Mota-Hernandez *et al.* utilized such scoring system to assess the severity of diarrhea in children caused by rotavirus infection with different genotypes of VP4 gene, unidentified P type (new P type) and type P[8] [21]. Zhang *et al*. also stated that rotavirus diarrhea caused by G1 was associated with higher severity scores than diarrhea caused by G3 in their study of rotavirus genotypes and disease severity [27]. This scoring system was also used to assess the relationship between rotavirus genotypes and the severity of diarrhea in a population of children in India [28].

Host factors are also very important in infections. Immune status and nutrition affect host responses to bacterial and viral pathogens [29,30]. Compared to bacteria, the infectious potential of viruses in humans is comparative and the host’s defenses are essential for prevention [31,32]. Environmental issues such as drinking water, hand washing and family income also play a role in the prevention of infectious disease [33].

In this study, we sought the correlation between rotavirus genotypes, clinical factors and degree of severity of acute diarrhea in children under 5 years old in Surabaya, Indonesia.

## Methods

### Patients

For one year surveillance, the data and the stool samples were gathered from April to December 2013. A total of 220 stool specimens were collected from the inpatients diagnosed with acute diarrhea in the Department of Pediatrics, Soetomo Hospital, Surabaya, Indonesia. The stools samples were from the consecutive patients in this studied time. The children with chronic diarrheas were excluded from the study.

PCR examination was done in 88 patients who had positive immunochromatography tests for virus detection. The study was approved by the Health Research Ethics Committee of Soetomo Hospital. Research subjects were all patients hospitalized in the Gastroenterology Ward in Department of Pediatrics of Soetomo Hospital. Inclusion criteria: children between 1–60 months with positive results from rotavirus antigen examination. Patients were excluded from the study if they were treated with antibiotics, probiotics and zinc before hospitalization, or had a history of rotavirus immunization or were in an immunocompromized condition (eg. from long term steroid therapy or cytostatic agents), who refused to continue the study. All study subjects underwent a physical examination, and parents or caretakers filled out a questionnaire. Stool specimens were collected and sent to the laboratory in Institute of Tropical Disease within 24 hours for bacterial culture and rotavirus molecular biology studies.

### Diagnoses of rotavirus

Stool rotavirus was examined by immunochromatographic methods (BD Rota/AdenoExamine™ kit), BD Biosciences, San Jose, CA. Stool samples were selected for rotavirus genotyping by a two-step RT-PCR method and included to the study by randomization from rotavirus positive stool samples [34]. The viral RNA was extracted using Viral Nucleic Acid Extraction Kit II (Geneaid Biotech Ltd., New Taipei) according to the manufacturer’s protocol. Two-step reverse transcription-PCR (RT-PCR) was carried out with the ThermoScript™ RT-PCR system to synthesize cDNA corresponding to the genomic segments encoding VP7 and VP4 with the protocol recommendation by the company. Used PCR primers are shown in Table 1. As to G typing, the primers VP7F and VP7R were used in RT-PCR (30 cycles) for an 881-bp VP7 gene segment. Then, VP7F was used in PCR (30 cycles) with type-specific such primers as aBT1 (G1), aCT-2 (G2), G3 (G3), aDT4 (G4), aAT8 (G8) and aFT9 (G9). For P-typing, the primers VP4F and VP4R were used in RT-PCR (30 cycles) for a 663-bp fragment and VP4F was then used in PCR (30 cycles) with such type-specific primers as 1T-1D (P[8]), 2T-1 (P[4]), 3T-1(P[6]), 4T-1 (P[9]) and 5T-1 (P[10]). Primers corresponding to the VP7 and VP4 genes for rotavirus genotyping were obtained from Integrated DNA Technologies, Singapore. All PCR products were separated in 2% agarose gel with a 50-bp DNA ladder as a standard marker and visualized by UV light after ethidium bromide-staining. All these procedures were performed according to the methods of WHO manuals [35].

**Table 1:** **Primers correspond to VP7 and VP4 genes for rotavirus genotyping**

**Primer**		**Sequence (5′ – 3′)**	**Position**	**PCR product (bp)**
G-typing (VP7) first amplification				
	VP7-F	ATG TAT GGT ATT GAA TAT ACC AC	nt 51-71	881
	VP7-R	AAC TTG CCA CCA TTT TTT CC	nt 914-932	
G-typing (VP7) second amplification				
G1	aBT1	CAA GTA CTC AAA TCA ATG ATG G	nt 314-335	618
G2	aCT2	CAA TGA TAT TAA CAC ATT TTC TGT G	nt 411-435	521
G3	mG3	ACG AAC TCA ACA CGA GAG G	nt 250-269	682
G4	aDT4	CGT TTC TGG TGA GGA GTT G	nt 480-498	452
G8	aAT8	GTC ACA CCA TTT GTA AAT TCG	nt 178-198	754
G9	mG9	CTT GAT GTG ACT AYA AAT AC	nt 757-776	179
P-typing (VP4) first amplification				
	VP4-F	TAT GCT CCA GTN AAT TGG	nt 132-149	663
	VP4-R	ATT GCA TTT CTT TCC ATA ATG	nt 775-795	
P-typing (VP4) second amplification				
P[4]	2T-1	CTA TTG TTA GAG GTT AGA GTC	nt 474–494	362
P[6]	3T-1	TGT TGA TTA GTT GGA TTC AA	nt 259–278	146
P[8]	1T-1	TCT ACT TGG ATA ACG TGC	nt 339 –356	224
P[9]	4T-1	TGA GAC ATG CAA TTG GAC	nt 385–402	270
P[10]	5T-1	ATC ATA GTT AGT AGT CGG	nt 575–594	462

*Refere*nces: [35].

### Detection of co-infection with norovirus: xTAG® GPP assay

The xTAG® Gastrointestinal Pathogen Panel (xTAG®GPP) (Luminex corporation, Austin, TX) is a qualitative multiplex PCR assay to detect simultaneously 15 different pathogens including norovirus in human stool samples. Solid stool (100-150 μg), or 100 μl of liquid stool was added to a Bertin SK38 Soil Mix Bead tube (BioAmerica Inc., Miami, FL) containing 900 μl of NucliSENS lysis buffer (bioMérieux, Durham, NC) [36]. RNA isolation for the xTAG® GPP assay was performed on the QIAamp MinElute Virus Spin kit (Qiagen, Hilden, Germany) [37]. A multiplex PCR was prepared by adding the xTAG GPP Primer Mix (Luminex corporation). PCR amplification cycling parameters were a reverse transcription (RT) step at 53°C for 20 min followed by an enzyme activation step at 95°C for 15 min and then 38 cycles of 95°C for 30 s, 58°C for 30 s and 72°C for 30 s, and followed by a final elongation step at 72°C for 2 min [38]. Following the incubation of the RT-PCR products with the xTAG GPP bead mix, streptavidin-PE conjugate and xTAG reporter buffer (Luminex corporation), the mixture was allowed to hybridize in the thermocycler for 3 min at 63°C followed by 45 min at 45°C. Data acquisition and analysis was performed on the MAGPIX instrument using xPONENT 4.2 software: positive and negative results were linked to a ratio between the target median fluorescence intensity and the threshold. An internal control (bacteriophage MS2) was included in each specimen [39].

### Detection of co-infection with bacteria

Regarding the method of detection of co-infection with bacteria, we performed conventional bacterial culture method using all the stool samples according to the previous work [35,40-44].

### Clinical factors

Clinical factors that were associated with the rotavirus genotyping included patient’s age (months), parents’ education, source of drinking water, duration of diarrhea before hospitalization, type of diarrhea, family income (monthly), type of milk, frequency of diarrhea, cough, fever, antibiotic administration, body temperature, nutritional state, co-bacterial infection, co-infection of upper respiratory tract infection (URTI), results of bacterial culture, and co-viral infection.

### Severity of diarrhea

The severity of diarrheal disease is related to clinical symptoms such as vomiting, the frequency of diarrhea, increase in body temperature, dehydration requiring intravenous rehydration fluid administration, the need for hospitalization, and the number of days the patient has been suffering from diarrhea. The diagnosis of the severity of diarrhea in this study was done at the time of recruitment of study subjects, which was when the diarrhea patients were admitted to the hospital. The severity of diarrhea was measured through the scoring system designed by Ruuska and Vesikari, which was tested for reliability and internal validity by Freedman, with Cronbach’s α = 0.7 (Table 2).

**Table 2:** **Parameters and Vesikari clinical scoring system**

	**Score**			
Parameters	0	1	2	3
Diarrhea				
Frequency, per days		1-3	4-5	≥6
Duration (days)		1-4	5	≥6
Vomiting				
Frequency, per days	0	1	2-4	≥5
Duration (days)		1	2	≥3
Body temperature (°C)	<37.0	37.1-38.4	38.5-38.9	≥39.0
Dehydration	None (<5%)	5-10%	>10%	
Treatment	None	ORS	Hospitalized	

### Statistical analysis

Statistical analysis was performed using SPSS statistics 17.0 (WinWrap® Basic, Polar Engineering and Consulting). Descriptive statistics using means, medians, standard deviation and confidence intervals were performed on all variables where appropriate. Inferential analysis was performed using the chi square and t-test.

## Results

Eighty-eight samples positive for rotavirus out of the 220 fecal samples were analyzed using immunochromatographic methods.

### Patient characteristics

Patient characteristics are shown in Tables 3 and 4. In brief, 51 (58%) were male and 37 (42%) were female. The age (months) was 14.6 ± 11.0. The most prevalent family income was US$ 90–179 in 41 patients (46.6%), followed by US$ 45–89 in 31 patients (35.2%). The prevalent source of drinking water was mineral water in 50 patients (56.8%), followed by tap water in 33 patients (37.5%). Frequency of diarrhea was 6.22 ± 3.85 times a day. Cough was seen in 25 patients (28.4%) and fever was seen in 72 patients (81.8%). Type of milk was predominantly formula milk (48.9%) followed by breast feeding + formula milk (31.8%). Antibiotics were administered in 20 patients (22.7%). Nutrition status was assessed as normal (55.7%), wasted (17.0%) and severely wasted (21.6%).

**Table 3:** **Overview of clinical characteristics**

**Variable**	**N**	**%**
Age (months)		
1-5	8	9.1
6-23	71	80.7
24-60	9	10.2
Sex		
Male	51	58
Female	37	42
Nutritional state		
Severly wasted	19	21.6
Wasted	15	17.0
Normal	50	56.9
Overweight	4	4.5
Maternal education		
Low	11	12.5
Mid	65	73.9
High	12	13.6
Breastfeeding state		
Never	13	14.8
<6 months	42	47.7
6-12 months	15	17.0
>12 months	18	20.5

**Table 4:** **Clinical manifestations**

**Variable**	**N**	**%**	**Mean**	**SD**
Duration of diarrhea (hours)			55.54	30.73
Diarrhea frequency over 24 hours (each)			6.26	3.85
Vomiting	67	77.0		
Duration of vomiting (days)			1.82	1.10
Vomiting frequency over 24 hours (each)			4.76	3.64
Temperature (°C)			37.45	0.73
Type of diarrhea				
Watery	68	77.3		
Loose	19	21.6		
Bloody	0	0		
Mucous	1	1.1		
With URTI* (cough/cold)	25	28.7		
Dehydration				
No dehydration	0	0		
Some dehydration	78	88.6		
Severe dehydration	10	11.4		
Coinfections	64	72.7		
Concomitant pathogens				
*Eschelichia coli*	37	42.5		
*Klebsiella* spp.	24	27.6		
*Enterobacter* spp.	3	3.4		

*URTI: Upper respiratory tract infection.

### Co- infection pathogens

Co-bacterial infection was seen in 64 patients (72.7%). The most prevalent bacteria was *Escherichia coli* (37/64, 57.8%) followed by *Klebsiella* (24/64, 37.5%). Co-viral infection was seen in 20 patients (22.7%) with rota + noro seen in 17 patients (85.0%) and rota + adeno + noro seen in 3 patients (15.0%).

### Rotavirus typing

**Figure 1:**
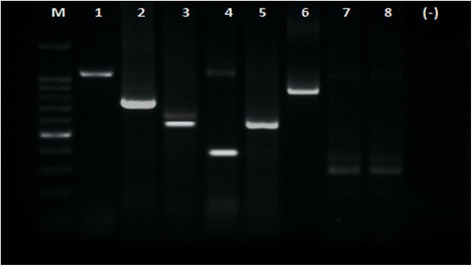
**Gel electrophoreses from stool samples.** PCR amplification result for G-type. Lane M: DNA Step Ladder marker, lane 1 (881 bp, full length VP7 RT-PCR product: positive control), lane 2 (G1, 618 bp), lane 4 (G4, 452 bp), lane 5 (G2, 521 bp), lane 6 (G3, 682 bp), lanes 7 and 8 (G9, 179 bp). (−): negative control.

**Figure 2:**
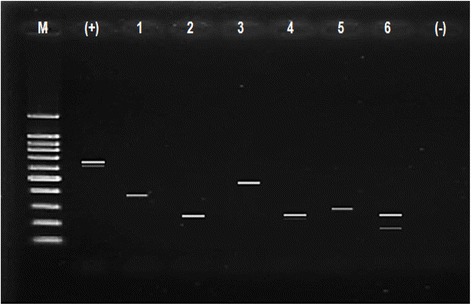
**Gel electrophoreses from stool samples.** PCR amplification result for P-type. Lane M (DNA Step Ladder), lane (+): positive control (663 bp, full length VP4 gene), lanes 1 (P[4], 362 bp), lanes 2 and 4 (P[8], 224 bp), lane 3 (P[10], 462 bp), lane 5 (P[9], 270 bp) and lane 6 (P[6]P[8], 146 bp and 224 bp). Lane (−): negative control.

**Figure 3:**
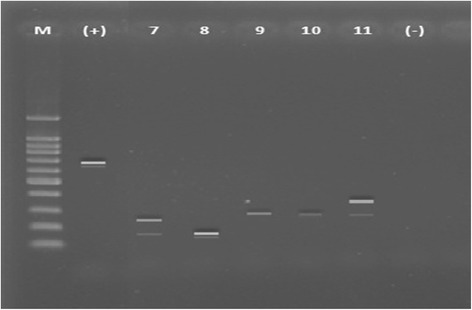
**Gel electrophoreses from stool samples.** PCR amplification result for P-type. Lane M (DNA Step Ladder), lane (+): positive control (663 bp, full length VP4 gene), lane 7 (P[6]P[8], 146 bp and 224 bp), lane 8 (P[6], 146 bp) , lane 9 and 10 (P[9], 270 bp), and lane 11 (P[4]P[9], 362 bp and 270 bp). Lanes (−): negative control.

Rotavirus typing in VP7 and VP4 was classified as common (G1, G2, G3, G4 and G9 (VP7) and P[4], P[6] and P[8] (VP4)) and uncommon (others) typing. Common typing was detected in 68.2% in VP7 and in 85.2% in VP4. The predominant VP7 genotyping (G type) was G2 (31.8%), followed by G1 (29.5%), G9 (11.4%), G4G9 (8.0%), and G1G2 (4.6%). As to VP4 genotyping (P typing), P[4] was predominant (31.8%), followed by P[6] (27.3%), P[8] (26.1%), P[9] (4.5%) and P[6]P[8] (4.5%). The typing was confirmed by PCR (Figures 1, 2 and 3). Combinations of G and P typing were classified as common (G1P[6], G1P[8], G2P[4], G2P[6], G3P[6], G4P[6] and G4P[8]) or uncommon types as well. Common typing was detected in 52.3% (Table 5). Uncommon typing data are also shown in Table 5. The predominant common type was G2P[4] (19.3%), followed by G1P[6] (12.5%) and G1P[8] (11.4%). and the predominant uncommon types were G9P[4] (4.6%) and G1P[4] (4.5%).

**Table 5:** **Common and uncommon combination genotyping (G-type and P-type)**

**Common genotyping**			**N**	**%**	
G1P[8]			10	11.4	
G1P[6]			11	12.5	
G2P[4]			17	19.3	
G2P[6]			3	3.4	
G3P[6]			2	2.3	
G4P[6]			2	2.3	
G4P[8]			1	1.1	
Subtotal			46	52.3	
**Uncommon genotyping**	**N**	**%**		**N**	**%**
G1P[4]	4	4.5	G2P[4]P[8]	1	1.1
G1G2P[6]	2	2.3	G2P[6]P[8]	2	2.3
G1G2P[8]	2	2.3	G4P[4]	1	1.1
G1G4G9P[8]	1	1.1	G4G9P[4]	1	1.1
G1G4P[4]	1	1.1	G4G9P[6]	1	1.1
G1G4P[6]	1	1.1	G4G9P[8]	3	3.4
G1G9P[8]	1	1.1	G4G9P[10]	1	1.1
G1P[4]P[8]	1	1.1	G4G9P[4]P[9]	1	1.1
G2P[8]	2	2.3	G9P[4]	4	4.5
G2P[9]	2	2.3	G9P[6]	2	2.3
G2G4P[8]	1	1.1	G9P[8]	2	2.3
G2G4P[9]	2	2.3	G9P[6]P[8]	2	2.3
G2P[10]	1	1.1			
Subtotal			n = 42		(47.7)

Common genotype combination groups included 7 genotype variations and uncommon genotype groups included 25 genotype variations. G2P[4] genotypes predominated among the common genotype groups. The highest number of uncommon genotype groups found were G1P[4] and G9P[4]. Twenty-five variations of uncommon genotype combinations were found in small quantities (Table 5).

The total severity score for uncommon GP-type and uncommon G-type rotaviruses was higher than for common GP-type and common G-type with statistically significant differences. P-types showed no significantly differences in total severity score between common and uncommon genotype groups (Table 6).

**Table 6:** **Correlation of total severity score to rotavirus genotypes**

**Variable**	**Type**	**N**	**Mean total score**	**SD**	**p-values**
GP-type	Uncommon	41	11.56	1.58	**0.000**
	Common	47	9.06	1.82	
G-type	Uncommon	28	12.00	1.41	**0.000**
	Common	60	9.40	1.87	
P-type	Uncommon	13	10.85	1.73	0.256*
	Common	75	10.12	2.17	

*Mann–Whitney test, p < 0.05 (significant); bold: statistically significant.

### Rotavirus typing and diarrhea

G3, G4 and G9 were significantly correlated with severe diarrhea (p = 0.009) in our multivariate analyses and with frequency of diarrhea (>10 times a day) (p = 0.045) in univariate analyses, but there was no significant correlation between P typing and severity of diarrhea. Regarding the combination of G and P typing, G2P[4] was significantly correlated with severe diarrhea in multivariate analyses (p = 0.029) (Tables 7, 8 and 9).

**Table 7:** **Correlation of severity of diarrhea with genotypes**

		***Univariate***		***Multivariate***	
**Genotype**	**N (%)**	**OR**	***p***	**OR**	***p***
G1	34 (38.6)	2.08	0.100		
G2	33 (37.5)	**0.25**	**0.004**	0.36	0.089
G3, G4, G9	30 (34.1)	**5.22**	**0.001**	**10.75**	**0.009**
P4	31 (35.2)	0.85	0.722		
P6	28 (31.8)	0.61	0.281		
P8	29 (33.0)	1.93	0.154		
P9, P10	7 ( 8.0)	1.51	0.605		

Bold: statistically significant. OR: odds ratio.

**Table 8:** **Correlation of severity of diarrhea with combination genotypes**

		***Univariable***		***Multivariable***	
**Genotype**	**N (%)**	**OR**	***p***	**OR**	***p***
G1P4	6 (6.8)	6.08	0.106		
G1P6	14 (15.9)	1.11	0.853		
G1P8	15 (17.0)	1.31	0.634		
G1P9, G1P10	0 ( 0.0)				
G2P4	18 (20.5)	**0.94**	**0.003**	**0.95**	**0.029**
G2P6	7 ( 8.0)	0.41	0.303		
G2P8	8 ( 9.1)	3.67	0.125		
G2P9, G2P10	5 ( 5.7)	0.72	0.723		
G3P4, G4P4, G9P4	10 (11.4)	**5.18**	**0.046**	9.22	0.087
G3P6, G4P6, G9P6	10 (11.4)	1.11	0.879		
G3P8, G4P8, G9P8	11 (12.5)	3.37	0.089		
G3P9, G3P10, G4P9, G4P10, G9P9, G9P10	4 (4.5)	9.68	0.134		

Bold: statistically significant. OR: odds ratio.

**Table 9:** **Correlation of G3, G4, and G9 genotypes with risk factors except for severity of diarrhea**

		***Univariate***	
**Risk factor**	**N (%)**	**OR**	***p***
Gender (female)	37 (42.0)	1.08	0.860
Age >10 years	56 (63.6)	1.53	0.374
Co-infection with other virus	20 (22.7)	0.40	0.139
Temperature (>38°C)	26 (29.5)	1.31	0.576
Co-infection with bacteria	64 (72.7)	0.64	0.360
Cough	26 (29.5)	1.31	0.576
Type of diarrhea	68 (77.3)	1.74	0.333
Frequency of diarrhea (>10 times a day)	16 (18.2)	**3.12**	**0.045**
Hand-washing	3 ( 3.4)	**-**	**-**
Mother hand-washing	8 ( 9.1)	1.18	0.831
Vomiting	68 (77.3)	1.27	0.661
Lavatory	11 (12.5)	1.73	0.399
Fever	72 (81.8)	1.17	0.791

Bold: statistically significant.

## Discussion

Rotavirus infection is a common cause of pediatric acute diarrhea especially in developing countries [4-6], and the most common cause of non-bacterial gastroenteritis in children not only in developing countries but also in developed countries. The positive rate for rotavirus varies between countries and regions, and the different detection rates may be explained by different study conditions, such as the season of the year or sampling methods.

We characterized the VP7 (G genotype) and VP4 (P genotype) gene segments in this study along with patients’ backgrounds and symptoms, and then identified the most common rotavirus combinations in our study. The rotavirus genotypes identified were very diverse. Genotypic variations were classified as common and uncommon based on descriptions by Kobayashi *et al.* [8]. We found genotype G2P[4] to be the dominant genotype with an overall prevalence of 19.3% in the subjects studied. As mentioned above, G1P[8], G2P[4], G3P[8], and G4P[8] were the four most common dominant genotypes in the world previously. The prevalence of each genotype was 52%, 11%, 3%, and 8% [10,11] respectively. These results differ from the studies by Sunarto *et al.* who found G1P[6] genotype as the dominant genotype [17]. Putnam *et al*. identified G2P[4] as the predominant genotype in the common genotype group, followed by G1P[6] [14]. Assuming a similarity in identification techniques, the difference in results may indicate that genotype prevalence in circulating rotaviruses may change periodically subject to natural fluctuations as reported by Gentsch *et al*. It is recommended that rotavirus vaccination programs conduct surveillance on an ongoing basis, including before and after implementation of vaccination programs [45]. Tate *et al.* suggested that the genotypes found to be circulating before the vaccination period should be used as a reference for the composition of the rotavirus vaccine, in order to produce vaccines with high efficacy [46]. Monitoring of genotypic variability after rotavirus vaccination makes it possible to identify genotypes that evolve in response to the selection pressure of the vaccine to emerge as new phenotypes that are immune to older vaccines. The emergence of immune genotypes has to be watched for and accommodated in the design of the next vaccines [31].

In this study, 64 subjects (72.7%) were found to be co-infected with bacteria. The commonest pathogen found concomitantly with rotavirus infection was *E. coli*. A systematic review by Grimprel *et al.* drawing on 173 English language journals from 1989 to 2006 reporting research in various countries around the world stated that the co-occurrence of diarrhea pathogen in the studied populations ranged from 0.3%–45.5%, and this range may reflect local epidemiology, economic development, and hygiene conditions [32]. A cohort study by Souza *et al.* in 154 children under 5 years suffering from acute diarrhea in Brazil revealed that 16.2% of rotavirus infections had co-infection with bacteria, particularly pathogenic *E. coli* [31].

Genotyping results were significantly associated with the severity of diarrhea using Vesikari symptom scores. In multivariate analyses, G3, G4 and G9 correlated significantly with severe diarrhea and frequent diarrhea and G2P[4] showed a significant correlation with severity of diarrhea. Further examination with longer periods of surveillance should be continued to monitor trends in this disease.

We would like to emphasize the limitations of this study. First, the number of cases and the study period are not enough to draw definitive conclusions. Nonetheless, these data are of importance since the studies in eastern Indonesia are lacking despite the prevalence of this disease in this region and its standing as a social issue in Indonesia. Secondly, seasonal data on viral isolation may show the trends in the spread of pathogens. Long-term regional surveillance is needed to overcome these limitations.

## Conclusions

The present study reports the current situation for acute diarrhea caused by rotavirus in infants or younger children in east Indonesia, Surabaya. Genotype G2P[4] has the highest prevalence, and G3, G4 and G9 and G2P[4] combination genotype were found to be associated with severe and frequent diarrhea. Further long-term studies as well as surveillance programs are necessary for overcoming rotaviral disease.
